# Hot and Cold Smells: Odor-Temperature Associations across Cultures

**DOI:** 10.3389/fpsyg.2017.01373

**Published:** 2017-08-10

**Authors:** Ewelina Wnuk, Josje M. de Valk, John L. A. Huisman, Asifa Majid

**Affiliations:** ^1^Centre for Language Studies, Radboud University Nijmegen, Netherlands; ^2^Max Planck Institute for Psycholinguistics Nijmegen, Netherlands; ^3^Donders Institute for Brain, Cognition and Behaviour, Radboud University Nijmegen, Netherlands

**Keywords:** cross-modal associations, olfaction, temperature, cross-cultural, cross-linguistic

## Abstract

It is often assumed odors are associated with hot and cold temperature, since odor processing may trigger thermal sensations, such as coolness in the case of mint. It is unknown, however, whether people make consistent temperature associations for a variety of everyday odors, and, if so, what determines them. Previous work investigating the bases of cross-modal associations suggests a number of possibilities, including universal forces (e.g., perception), as well as culture-specific forces (e.g., language and cultural beliefs). In this study, we examined odor-temperature associations in three cultures—Maniq (*N* = 11), Thai (*N* = 24), and Dutch (*N* = 24)—who differ with respect to their cultural preoccupation with odors, their odor lexicons, and their beliefs about the relationship of odors (and odor objects) to temperature. Participants matched 15 odors to temperature by touching cups filled with hot or cold water, and described the odors in their native language. The results showed no consistent associations among the Maniq, and only a handful of consistent associations between odor and temperature among the Thai and Dutch. The consistent associations differed across the two groups, arguing against their universality. Further analysis revealed cross-modal associations could not be explained by language, but could be the result of cultural beliefs.

## Introduction

Sniffing mint brings a cool refreshing scent to our noses, while the fragrance of chili peppers may be experienced as hot. In our daily life, we seem to experience odors as having temperature. Such sensations are due to the stimulation of the thermoreceptors within the trigeminal nerve, a sensory system responsible for tactile perception triggered by odors. Trigeminal perception is sometimes referred to as the “feel” of a smell (Herz, 2001), and together with olfactory perception forms part of our integrated experience of an odor, contributing not only to the sensations of temperature, but also touch, pressure, and pain (Hummel and Livermore, 2002).

The association between odor and temperature is also reflected in language. Some odors, for instance, are spoken of as being “cool” or “hot,” and although in English these are not fully conventionalized odor descriptors, a corpus search reveals they are occasionally used to describe smells, e.g., “the hot smell of plum brandy,” “the cool smell of the pine needles” (The Corpus of Contemporary American English; Davies, 2008). In some languages, however, a combination of both olfactory and thermal meaning under a single term is fully lexicalized, e.g., *fîk/vîk* in Lushai, spoken in India, can refer to pungent smells as well as biting cold (Lorrain, 1940, pp. 137, 551), *k^h^𝜀n2* in Shan, spoken in Burma, can describe strong heat (e.g., of rays of sun), as well as an overpowering, stifling smell (e.g., of liquor) (Sao Tern Moeng, 1995, p. 782).

Despite the prominent link between smell and temperature and smell-trigeminal interactions in foods (Labbe et al., 2008), there is little research directly testing temperature associations for everyday odors. For example, a recent review on cross-modal correspondences involving odors (Deroy et al., 2013) acknowledges the existence of odor-temperature associations, but does not mention any studies directly testing how these perceptual qualities are linked. In contrast, there have been numerous studies examining odor–color associations (Gilbert et al., 1996; Demattè et al., 2006; Spector and Maurer, 2012; Stevenson et al., 2012) and temperature-color associations (Ho et al., 2014a,b). These studies have emphasized the perceptual bases of these associations, but a recent study showed language can play an important role too (de Valk et al., 2016).

De Valk et al. (2016) conducted a cross-cultural study examining odor–color associations in three distinct cultures: the Maniq, Thai, and Dutch. These groups represent a spectrum in terms of how important olfaction is in their cultures and languages. For example, the Maniq and the Thai have elaborate vocabularies of abstract smell terms, whereas the Dutch have a much more impoverished language for olfaction, and refer to the sources of odors instead (e.g., *It smells like banana*). Participants were tested with a range of odors and asked to associate each with a color. De Valk et al. (2016) found across cultures, when participants used source-based terms (i.e., words naming odor objects, such as *banana*), their color choices reflected the source more often than when they used abstract smell terms (i.e., words describing smell qualities, such as *musty*). This suggests language plays an important mediating role for odor–color associations.

Similar questions can be asked about odor-temperature associations: Do they exist, and, if so, what is their basis? Insights from trigeminal perception indicate they could be shaped by human physiology. On the other hand, our previous cross-cultural work (de Valk et al., 2016) raises the possibility that culture-specific factors could be involved as well, especially since the three cultures differ markedly in how culturally and lexically elaborated olfaction is in each of them. However, since this is a largely unexplored area where no previous cross-cultural study has been undertaken, we do not favor a particular hypothesis, and consider a broad range of possibilities.

In order to see whether culture-specific factors could mediate the associations, we follow the procedure in de Valk et al. (2016). There are specific beliefs that we explore: (1) a belief embedded in the indigenous knowledge system of the Maniq linking smell terms and temperature, and (2) beliefs embedded in popular medical theory among the Thai linking odor objects and temperature.

The Maniq are a small nomadic community of rainforest hunter-gatherers living in southern Thailand. They speak a language with a rich vocabulary of abstract smell terms and have intricate cultural beliefs relating to odors (Wnuk and Majid, 2014). Abstract smell terms are words dedicated to smells that describe odor quality instead of referring to specific sources. For example, the term *lsp∂s* denotes a fragrant smell quality characteristic of a wide range of different objects (e.g., wild yams, bearcats, medicinal plants, various trees, and fruit). Crucially for the present study, the Maniq explicitly associate the quality described as *lsp∂s* with coolness (Wnuk, 2016, pp. 45–47), as the Maniq believe *lsp∂s*-smelling items have cooling properties. Thus, if Maniq indeed form consistent odor-temperature associations based on such beliefs, all odors described as *lsp∂s* should be consistently linked to cool temperature, irrespective of the specific odor object.

This contrasts with links via objects. These kinds of links are likely to be found in cultures which explicitly associate objects with specific temperatures, such as in Thai culture. According to some traditional beliefs in Thailand, many foods and herbs can be classified into hot and cold categories (Van Esterik, 1988; Salguero, 2003). These beliefs derive from the humoral theory of medicine, popularized by Hippocrates and Galen, and found in many cultures throughout the world (Foster, 1979; Messer, 1984; Anderson, 1987). For example, in Thailand garlic and most meats are considered hot, while jasmine tea and lotus root are cool (Salguero, 2003, p. 32). If such beliefs are used as basis of odor-temperature associations, we would expect objects to play a mediating role for associations, e.g., the smell of garlic would be associated with hot temperature because garlic is considered to be a “hot food.”

Yet another possibility is that odor-temperature associations are not dependent on cultural beliefs, but are universal, perhaps due to shared physiology. Laska (2001) found that menthol (peppermint) and cineole (eucalyptus) were consistently matched with a temperature term (*cool*). This is due to the trigeminal stimulation evoked by these odors. This hypothesis would predict similar results for all people, irrespective of whether people have beliefs about odor-temperature correspondences. If odors are invariably tied to a cool or hot feeling, everyone should have the same odor-temperature associations. Our study therefore included Dutch participants, who do not have an explicit humoral-based theory about food and temperature, and for whom no *a priori* beliefs about odor-temperature associations have been reported.

## Materials and Methods

### Participants

We tested 24 Thai speakers (*M*_age_ = 21; 19 female) recruited at the Ubon Ratchathani University and Kasetsart University, 24 Dutch speakers (*M*_age_ = 26; 18 female) participants recruited at the Radboud University, and 11 Maniq speakers (*M*_age_ = 41; 5 female) recruited at a forest campsite in the area of Manang district, Satun. The sample size of the Thai and Dutch was based on earlier studies on cross-modal correspondences which test 20–30 participants (e.g., Demattè et al., 2006; Stevenson and Oaten, 2008; de Valk et al., 2016). There were fewer Maniq participants because of practical difficulties of accessing large numbers of people in this remote community. All participants provided informed consent and were given monetary compensation, or equivalent, for participation. The research was approved by the Ethics Assessment Committee of Radboud University.

### Stimuli

Odor stimuli were real objects commonly ingested in the Netherlands (mustard, licorice, red wine, peanut butter, and cheese), Thailand (dried durian, shrimp paste, coconut milk, galangal, and fermented petai beans), or in both countries (banana, tobacco, cooked rice, garlic, and canned fish). We could not include the most salient *lsp∂s*-smelling items since they are highly culture-specific objects (e.g., bearcats, *Dioscorea* wild yams) that are difficult to reproduce systematically. However, we chose items, which based on our previous investigation (Wnuk and Majid, 2014) were likely to be classified as *lsp∂s*: namely, galangal, banana, cooked rice and dried durian. In addition, we included items that were considered hot or cold foods by Thai speakers. Some of these beliefs are fairly common. For instance, durian is widely known to be hot and banana cold, but there is also some variation in associations and level of expertise on the subject (Van Esterik, 1988; Salguero, 2003; Kumar et al., 2012), which we discovered after the fact. We presented real objects as stimuli. While real objects ensure greater ecological validity, there is less control over their perceptual characteristics. For each stimulus, we used a prescribed amount that was kept comparable across testing, e.g., 2 teaspoons of peanut butter, etc. We monitored the stimuli for freshness by checking all smells on average every 3 h and replacing the items which did not appear fresh with new ones. All objects were presented in opaque squeezy bottles so participants could not see what they were.

Temperature stimuli were two non-transparent white plastic cups with covers that were filled with either warm (boiled) or cold (iced) water. We used the same generic (unbranded) cups for hot and cold. They were 250 ml cups with a handle made of hard non-disposable plastic to prevent them from deforming under the pressure of heat. There was always a marked relative difference between the warm and cold cup throughout the experiment, although unavoidably the temperature in the cups changed slightly over the course of the experiment due to the practicalities of data collection in the field. In order to maintain this difference for all participants, freshly boiled water was added to the warm cup and ice was added to the cold cup in the breaks between participants.

### Design and Procedure

Participants first completed an odor-to-temperature matching task, in which they sniffed an odor and matched it to either warm or cold temperature by touching one of the two cups. The instruction delivered by the experimenter in the participants’ native language was a translation of the English sentence *“Please touch the cup whose temperature goes with this odor*.*”* No separate pre-test of olfactory ability was conducted, but participants were asked whether they had any general problems with their olfactory ability. No such problems were reported. The odor bottles were handled by the experimenter. The warm and cold cups were located within participants’ reach in front. The break between odors was the time needed to record the answer and exchange stimuli (minimum 25 s). Participants completed the odor-to-temperature matching task twice, with an average break of 2 h (range 1–4 h) in between. Odor stimuli were presented to all participants in a single fixed random order. The order was fixed within a task but differed across tasks.

After the second odor-temperature matching task, participants smelled the odors again and completed another task, in which they first answered the question *“How does it smell?”*, asked in their native language, and then rated odor familiarity. The rating was done on a simple 3-point scale (1 = unfamiliar, 2 = somewhat familiar, 3 = familiar) to make it more straightforward for the Maniq, who are not used to scales. The naming-plus-rating task always followed the matching tasks, so as not to influence the associations. All responses were noted down in an answer sheet and audio-recorded for later transcription.

## Results

### Do People Make Consistent Odor-Temperature Associations?

We first tested whether participants were consistent in how they associated odors to temperatures across trials. Each participant completed the odor temperature task twice. We considered a response to be consistent if the same temperature was chosen across both sessions. One Maniq participant completed the odor temperature task only once and was therefore excluded from analyses where data from both blocks was needed.

To test whether the associations were robust over time, i.e., whether participants chose the same temperature for an odor across the two sessions, binomial tests were performed (where chance is 0.5). Odor-temperature associations were consistent for Thai (*N* = 360, observed proportion = 0.70, *p* < 0.0001) and Dutch (*N* = 360, observed proportion = 0.74, *p* < 0.0001), but not for Maniq (*N* = 150, observed proportion = 0.53), *p* > 0.05.

**FIGURE 1:**
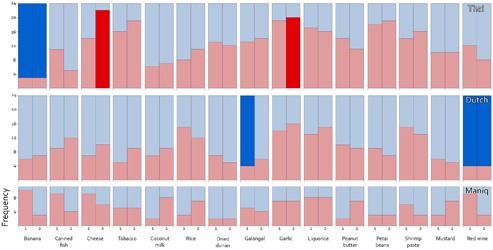
Temperature choices for odor, session, and language. *Y*-axis represents the frequency of hot (red) or cold (blue) choices. Binomial tests were conducted to test whether odors were significantly related to hot or cold temperature. Significant odor temperature associations are depicted by saturated colors. All other cases failed to reach significance.

Regardless of whether the temperature choice was consistent over time, we can ask which specific temperature was associated to each odor. Therefore we investigated the number of participants who chose hot/cold for each odor and compared this to the number expected by chance. Different temperatures may be chosen across sessions, therefore we examined block 1 and 2 separately. We applied a Bonferroni correction for the 15 odors we tested leading to a chance level of 0.003. There were few specific odor-temperature associations that were statistically significant according to this strict criterion. In the Thai group, banana was significantly related to cold (*N* = 24, observed proportion = 0.88, *p* < 0.001 for both Blocks 1 and 2), while cheese was related to hot (*N* = 24, observed proportion = 0.92, *p* < 0.001 Block 2), as was garlic (*N* = 24, observed proportion = 0.83, *p* < 0.002 Block 2), largely in line with the cultural beliefs among the Thai (see Discussion). In the Dutch group, wine was related to cold (*N* = 24, observed proportion = 0.83, *p* < 0.002 for both Blocks 1 and 2), and galangal was related to cold (*N* = 24, observed proportion = 0.83, *p* < 0.002 in Block 1). Finally, in the Maniq group none of the odors was significantly related to hot or cold. The Maniq participants also did not use the term *lsp∂s* in the task, so we could not verify the hypothesis related to that term. The results are displayed in **Figure 1**.

### What Predicts Odor-Temperature Associations?

There is some preliminary support that cultural beliefs might influence odor-temperature associations, but alone they explain only a small portion of the data, i.e., the associations in the Thai group. Another possibility is that, as in the case of color, cross-modal associations are shaped by language. Odor–color associations were found to reflect the source more often when odors were named with source-based terms (e.g., *banana*) compared to abstract terms (e.g., *musty*) (de Valk et al., 2016). To investigate if similar processes are at play for temperature associations, we examined participants’ odor descriptions and their consistency of odor-temperature associations.

We examined the main content descriptors from the odor naming task, disregarding modifiers. The responses were coded as “abstract” smell terms, “source-based,” and “other”. Abstract smell terms express a smell quality characteristic of a range of objects and do not refer to one particular object (as in *stink*). Source-based terms denote specific objects (e.g., *cheese, fruit*). The “other” category includes all other response types, including taste (e.g., *sweet*), texture (e.g., *soft*), and evaluative terms (e.g., *good*). The Dutch participants used predominantly source-based descriptors, followed by responses from the “other” category, and employed abstract terms such as *stinkend* ‘stinky’ and *muf* ‘musty’ in just a handful of cases (about 1% of the time). In contrast, the Thai and Maniq participants used abstract terms over a third of the time, similar to source-based terms, and employed “other” descriptors in a fourth of all cases. The most frequent abstract items included, for Thai, *hǒom* ‘to be fragrant, odoriferous, sweet smelling,’ *měn* ‘to smell bad, stink, be foul-smelling,’ and *chǔn* ‘to be strong (of odors), pungent (as the odor of strong tobacco),’ and for Maniq, *lsp∂s* ‘to smell fragrant, as of, e.g., medicinal plants, wild yams, bearcats, forest’, *haʔĩt* ‘to stink, as of, e.g., rotting carcass certain animals (e.g., bats), some wild yams (e.g., *Dioscorea daunea*)’, and *miʔbay
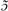
ϕ* ‘smell of, e.g., old shelters, soil, mushrooms, rotten leaves.’

Following the procedure in de Valk et al. (2016) we removed all “other” responses and first looked at source-based vs. abstract terms and their relationship to odor-temperature associations. We used mixed logit models (Jaeger, 2008) performed in R (R Core Team, 2013) using the lme4 package (Bates et al., 2014). Fixed factors were language (Thai, Dutch, Maniq), type of odor description (source-based, abstract), and familiarity rating (unfamiliar, somewhat familiar, familiar). Random intercepts were added for participants and items to control for individual variation. The dependent variable was temperature consistency, where the temperature was considered consistent if the same cup was chosen in both sessions, and inconsistent otherwise. There was no effect of language, β = 0.23, *SE* = 0.14, *z* = 1.67, *p* = 0.096. The type of description also did not predict consistency, β = 0.25, *SE* = 0.21, *z* = 1.18, *p* = 0.238, nor did familiarity of the odor, β = 0.21, *SE* = 0.13, *z* = 1.66, *p* = 0.097. So odor-temperature associations were not more consistent when the odor was described with a particular type of descriptor, or when it was judged as more familiar.

We also examined the effect of naming accuracy on temperature associations by comparing correct vs. incorrect responses. These analyses were conducted only with source-based descriptions—the only response type that can be judged to be either “correct” or “incorrect,” i.e., if the description is the name of the actual source object. Fixed factors were again language (Maniq, Thai, Dutch), accuracy of odor description (correct and incorrect), and familiarity rating (unfamiliar, somewhat familiar, and familiar), with random intercepts for participants and items. The dependent variable was again temperature match consistency. There was no effect of language, β = 0.01, *SE* = 0.19, *z* = 0.04, *p* = 0.971. The accuracy of the description also did not predict consistency, β = 0.30, *SE* = 0.26, *z* = 1.14, *p* = 0.253, nor did familiarity of the odor, β = 0.31, *SE* = 0.16, *z* = 1.90, *p* = 0.058. Once again, none of the language-related factors nor familiarity of the odor determined the consistency of odor-temperature associations.

## Discussion

The field of cross-modal correspondences has made significant advances over the last years (Spence, 2011; Deroy et al., 2013). However, so far there has been little empirical work directly testing the link between odor and temperature. Our study shows people are rarely consistent in their temperature associations for everyday odors. In cases in which they are consistent, different cultures display diverging patterns, suggesting these associations are not universal, but have a cultural basis instead.

We posited language and culture may play a significant role. Our results suggest to the contrary that language plays no role in odor-temperature associations. The type of description used for an odor did not predict consistency in odor-temperature matches, and it did not matter whether odors were named correctly. In particular, we predicted the Maniq would associate all smells described with the term *lsp∂s* with cool temperature. However, the Maniq barely described any smells as *lsp∂s* nor did they display consistent odor-temperature associations. We were therefore unable to evaluate the hypothesis. More generally, there was no evidence across any group that language plays a role in odor-temperature associations, unlike in odor-color associations (de Valk et al., 2016).

The lack of consistent associations among the Maniq is reminiscent of a similar finding for this group in another cross-modal experiment involving matching odors to colors (de Valk et al., 2016). This highlights the possibility that the Maniq may be adopting a distinct strategy in cross-modal matching, which could be related to environmental and cultural differences between them and the urbanized groups (cf. de Valk et al., 2016).

We also explored the possibility that Thai participants would base their odor-temperature choices on their beliefs about hot and cold foods: i.e., they would associate odors of objects considered as “hot foods” with hot temperature and odors of objects considered as “cold foods” with cold temperature. The specific associations we found (banana-cold, garlic-hot, and cheese-hot) were in line with cultural beliefs. Bananas—linked with cold in our task—are commonly regarded as a cooling food in Thailand (Kumar et al., 2012). Similarly, garlic—paired with hot in the task—is classified as a hot food (Salguero, 2003). These associations are not specific to the Thai culture, but exist in other Southeast Asian settings, e.g., in Malaysia (Manderson, 1981). There was one more significant odor-temperature association among the Thai which mapped cheese to hot, but there does not seem to be a specific prediction linked to cheese, since cheese is not a common food in Thailand. A clue as to why it was consistently associated with hot temperature comes from the naming data. The most frequent descriptor of cheese in the task was the word *bùt* ‘rotten’, occurring in expressions such as *nom bùt* ‘rotten milk’, *aahǎan bùt* ‘rotten food’, *khǒong bùt* ‘rotten thing’. It is this general quality, as well as similar qualities such as the state of being fermented, that Thai participants seem to link with heat (note that there was a trend for fermented petai beans to be linked with hot temperature too, see **Figure 1**). This suggests that—for novel or infrequent smell objects without a conventional temperature association—Thai participants might fall back on more general characteristics of the odor to determine the association. This suggests the mechanisms driving the associations might not be the same for all odors and might include object identity-based associations, as well as more abstract feature-based associations (Deroy et al., 2013).

There were several other odor-temperature associations we expected to find among Thai participants. In the case of coconut milk (linked to cold) and dried durian (linked to hot) there was a trend in the predicted direction, but the association was not significant. The belief about durian being a heat-inducing food is especially popular, so it is surprising the association with heat was not significant. We also predicted red wine would be linked to hot temperature, as alcohol is classified as a heat-inducing substance (Van Esterik, 1988), but in fact it was more commonly associated with cold. These cases suggest not all cultural beliefs are prominent or homogenous enough to lead to consistent associations (cf. Anderson, 1980; Manderson, 1981; Chang Ing, 2010). Finally, it is important to stress that while the hot/cold dichotomy is part of herbal medical theory in Thailand, it is especially well-known among healers and various specialists (Van Esterik, 1988), and might not be equally prominent among ordinary people, which could also explain the paucity of consistent associations.

Although we did not have culture-specific predictions about the Dutch, we found two consistent associations in this group: wine-cold (significant across two sessions) and galangal-cold (significant in one session). It is unclear what the underlying factors are in this case. They might reflect a kind of semantic congruency (i.e., smell of wine matched to typical temperature of wine) (cf. Deroy et al., 2013), but it remains to be explained why the effects apply only locally to a subset of stimuli. Despite the fact that both stimuli have strong trigeminal components—ethanol (wine), and galangal acetate (galangal)—it is unclear whether trigeminal stimulation could account for the particular associations. Based on previous studies examining these trigeminal components, it is questionable whether cold/cool would be the predicted association, since the descriptive profiles of these substances do not clearly suggest that (Yang and Eilerman, 1999; Laska, 2001).

### Limitations and Future Directions

While our results are suggestive of lack of universality of odor-temperature associations, there are some important caveats. Our stimuli were mostly food objects, not selected specifically to trigger strong trigeminal responses, but rather to represent a variety of everyday odors. Trigeminal perception can be evoked by almost all odors, but it only occurs if the odor is intense enough (Lundström et al., 2011). In addition, thermal sensations are only one type of a range of possible trigeminal reactions, and many odorants, e.g., alcohol, trigger a multitude of trigeminal sensations, including both cool and burning sensations (Laska, 2001). Future studies should extend this investigation by including a larger sample of trigeminal odorants with clear predictions, e.g., menthol (McKemy, 2007), and odors with clear predictions based on humoral theories. Similarly, future studies should incorporate pleasantness and intensity ratings. These were not included here due to concerns of participant fatigue (motivated mainly by the participants with no experience of formal schooling).

Based on the present results, if there are odor-temperature associations, it appears they might be small effects and our sample size may have been too small to detect these. Thus, for broad generalizability of findings, a larger number of participants need to be tested, and further cultures need to be included, to see whether the associations vary in line with cultural beliefs. Finally, because of the exploratory nature of our study we were not able to investigate possible underlying mechanisms. Further work could address this in more detail, and explore further the key properties of odor-temperature associations, e.g., whether they adhere to the predictions of the transitivity hypothesis (Deroy et al., 2013).

## Conclusion

To conclude, although the existence of odor-temperature associations is acknowledged (Laska, 2001; Deroy et al., 2013), explicit tests of temperature associations for everyday odors are still missing. This study suggests that some odors are associated to temperature and there is cultural variation in these associations. Cultural beliefs or culture-specific matching strategies may be important for shaping odor-temperature associations.

## Data Availability

The data (Wnuk et al., 2017) is publicly available at: https://doi.org/10.17026/dans-z3w-29j2.

## Author Contributions

EW, JH, and AM conceived and designed the study. EW and JH, ran the experiment and coded data. JdV and JH conducted statistical analyses under the supervision of AM. All have contributed to writing and editing the manuscript, with EW taking the lead.

## Conflict of Interest Statement

The authors declare that the research was conducted in the absence of any commercial or financial relationships that could be construed as a potential conflict of interest.
